# Template-Driven Knowledge Distillation for Compact and Accurate Periocular Biometrics Deep-Learning Models

**DOI:** 10.3390/s22051921

**Published:** 2022-03-01

**Authors:** Fadi Boutros, Naser Damer, Kiran Raja, Florian Kirchbuchner, Arjan Kuijper

**Affiliations:** 1 Fraunhofer Institute for Computer Graphics Research IGD, 64283 Darmstadt, Germany; florian.kirchbuchner@igd.fraunhofer.de (F.K.); arjan.kuijper@igd.fraunhofer.de (A.K.); 2 Mathematical and Applied Visual Computing, Technical University of Darmstadt (TU Darmstadt), 64289 Darmstadt, Germany; 3 The Norwegian Colour and Visual Computing Laboratory, Norwegian University of Science and Technology (NTNU), 2815 Gjøvik, Norway; kiran.raja@ntnu.no

**Keywords:** biometrics, knowledge distillation, periocular verification

## Abstract

This work addresses the challenge of building an accurate and generalizable periocular recognition model with a small number of learnable parameters. Deeper (larger) models are typically more capable of learning complex information. For this reason, knowledge distillation (kd) was previously proposed to carry this knowledge from a large model (teacher) into a small model (student). Conventional KD optimizes the student output to be similar to the teacher output (commonly classification output). In biometrics, comparison (verification) and storage operations are conducted on biometric templates, extracted from pre-classification layers. In this work, we propose a novel template-driven KD approach that optimizes the distillation process so that the student model learns to produce templates similar to those produced by the teacher model. We demonstrate our approach on intra- and cross-device periocular verification. Our results demonstrate the superiority of our proposed approach over a network trained without KD and networks trained with conventional (vanilla) KD. For example, the targeted small model achieved an equal error rate (EER) value of 22.2% on cross-device verification without KD. The same model achieved an EER of 21.9% with the conventional KD, and only 14.7% EER when using our proposed template-driven KD.

## 1. Introduction

Biometric deployment on smartphones and different embedded devices enables novel applications for identity verification or service personalization. Facial characteristics are one of the commonly used biometric traits for identity recognition. This is driven by the high user acceptance [1] and the non-invasive touchless capture process [2,3,4]. Moreover, the high performance achieved by the recent approaches proposed in the literature [5,6,7] led to face recognition gaining larger deployment ground. However, face occlusions due face masks, especially after the recent COVID-19 pandemic, present a challenge for face recognition systems [8]. Recently, several studies [9,10,11,12] evaluated the effect of wearing a face mask on face recognition performance. These studies [9,10,11] concluded that the verification performance of face-recognition solutions significantly degraded when the subject was wearing a face mask, compared to the case where their face was unmasked. This was followed by several efforts to enhance masked face recognition [13,14,15], including competitions where some of the submitted solutions proposed the use of the periocular recognition [16,17]. Periocular biometrics have a distinct advantage over facial biometrics when the face is largely occluded or when capturing a full face is less convenient than capturing the periocular region (e.g., a selfie on a smartphone or masked face recognition [11], while maintaining the touchless nature of face capture. It also carries other benefits; for example, perspective distortion affects the periocular region to a lower degree because the depth variation of the area is smaller than the that of the complete face [18]. Periocular recognition on smartphones and wearable devices has gained growing research attention, and with recent deep learning methods achieving superior accuracy and with applications in various domains have surfaced [19,20,21,22]. However, deploying large models based on deep learning on resource-critical consumer devices presents two main challenges. The first relates to the highly constrained computational requirements of these devices, requiring the models to be compact. Given the increased number of smart applications running on these devices, keeping the number of trainable parameters of any deep learning model to a minimum is essential for acceptance by customers against having applications that require gigabytes of memory. The second challenge is related to cross-device/cross-camera biometric performance, which often results in data with high variations. A small deep learning model for periocular recognition should be able to compensate for such variations in capture conditions. Learning complex image information such as identity is more realizable in relatively deeper models [23]. However, such models contain a large number of trainable parameters, as mentioned earlier. The post-training minimization of these complex models has been addressed mainly by parameter pruning, quantization, and knowledge distillation (KD). While the first two change the trained network structure, KD transfers its knowledge into a smaller (student) network. Conventional KD deployments optimize the distillation process so that the student network achieves similar output as the teacher (larger and trained) network.

Many biometric applications resort to deep learning models that are trained as identity classifiers. However, these models are used as template (feature) extractors by sampling pre-classification network layers [24,25]. This renders the recent proposals of deploying conventional KD on biometric networks [26] theoretically sub-optimal, as it teaches the less-complex student network to classify the identity correctly, rather than to extract an optimal (with respect to the teacher network) template.

The present work addresses this issue by proposing its main contribution, a novel template-driven KD for periocular recognition that transfers the optimal template extraction knowledge from a deeper network into a network with a significantly lower number of trainable parameters. The proposed approach is designed so that it can be adapted to any suitable student/teacher architecture combinations. We extensively demonstrate the applicability of our proposed approach for periocular verification, both for intra-device and cross-device scenarios, to show the effectiveness of our proposed template-driven KD. To demonstrate the generalizability of the proposed concept, we train our template-driven KD network with two different loss functions, mean squared error (MSE) loss and Cosine loss. Further, we compare the performance of the resulting student network with the same network when trained from scratch, as well as two different larger teacher networks to validate our assertion. Our results are further benchmarked against the conventional KD optimized for classification, consistently showing the benefits of our proposed solution. These achievements are demonstrated on a periocular dataset of 152 unique identities consisting of 6682 images captured with 2 different smartphones (iPhone 5S and Nokia Lumia 1020) to demonstrate generalizability across capture devices. We demonstrate that our template-driven KD solution successfully and consistently reduced the verification error rates under cross-device experimental settings. Our template-driven KD solution successfully reduced the cross-device false non-match rate (FNMR) at a false match rate (FMR) of 10% of the same efficient network structure from 39.45% when trained from scratch and 23.2% when conventional KD is applied, to 20.1%.

In the remainder of this article, we present a set of related works in Section 2 and the proposed approach along with baseline approaches in Section 3. We provide the details on the conducted experiments in Section 4 and the obtained results in Section 5. Finally, we present our conclusions in Section 6.

## 2. Related Work

Periocular recognition is preferred when capturing the full face consistently is a challenge [8,11,15], for instance when faces are occluded due to masks or clothing preferences. Park et al. [27] presented one of the first works proposing the use of the periocular region as a biometric trait captured under a controlled environment. Based on the approach of Park et al. [27], several subsequent works were proposed: Juefei-Xu et al. [28] utilized LBP to encode discrete transforms enabling a translation-robust descriptor. Mahalingam and Ricanek Jr. [29] proposed the use of multi-scale patch-based LBP feature descriptors. Ross et al. [30] presented a fusion-based schema to handle the variability in periocular input images. Recently, many approaches using deep learning have been proposed for periocular recognition [31,32,33]. Proenca et al. [31] trained convolutional neural networks (CNNs) to implicitly learn the region of interest (periocular area) without localizing the iris. Zhao and Kumar [33] proposed a semantics-assisted CNN (SCNN) model consisting of multiple CNN models for periocular recognition. Rattani et al. [32] evaluated off-the-shelf deep CNNs for periocular recognition and illustrated the benefit of fine-tuning CNNs.

As noted by Rattani et al. [32], the high variability between probe and gallery images, produced when the images are acquired using different devices, challenges the biometric performance. A generalized solution to such challenging data is needed to mitigate biometric performance loss, and some works have partially addressed this issue in recent years [20,34,35,36,37]. While previous deep-learning-based solutions addressed the problem to a certain degree, the computational complexity of the models is rather high, with many trainable parameters [34,37], and this specifically is not preferred in smartphone applications due to limited computation resources. Both models presented in [34,37] contain more than 12 million trainable parameters.

In order to address the constraints of compact model requirements, earlier works have minimized deep-learning-model size using mainly one of three methods, parametric pruning [38], quantization [39], and KD [40]. While the first two methods process a relatively large network into a smaller one, KD transfers the acquired knowledge learned by a larger network (teacher network) to a smaller one (student network). Common KD deployments optimize the distillation (teaching) process on the same network output that the teacher network used to optimize its own training, for example, classification accuracy [41].

While this seems a feasible solution, biometric applications need to extract identity information (e.g., feature representation or templates) in order to achieve better performance [24,42]. Performing conventional KD, optimized on the classification output, has recently been applied in the biometric domain [26,43]. This makes the distillation of the classification knowledge sub-optimal for a student network that will be deployed as a template extractor, as will be shown later. In this work, although the teacher network is trained to optimally classify identities, we distill the knowledge to achieve optimized template generation by the student network that, by itself, is too shallow to learn templates which are as optimal as those of its teacher.

## 3. Baseline Models and Proposed Approach

**Figure 1:**
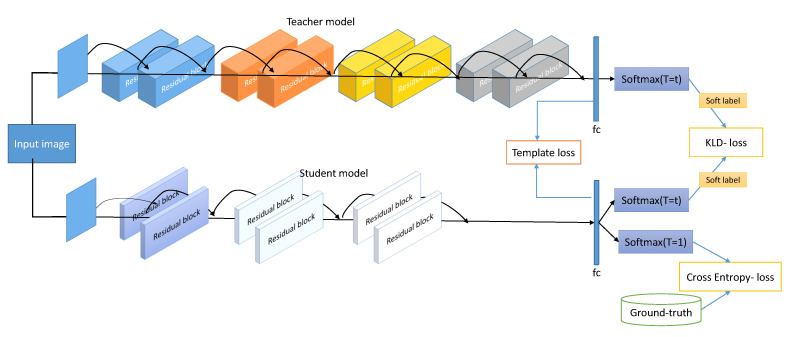
Overview of the proposed template-driven KD approach for periocular verification based on the ResNet architecture. Note that both the template loss and KD output loss contribute to the distillation process.

In this work we present a template-driven KD framework to enhance the accuracy and generalizability of a compact CNN model for periocular verification under a smartphone verification scenario. Figure 1 illustrates the proposed template-driven KD framework. In this work, we propose to train the student model to learn the logits and the template embedding from the teacher model. We achieved this by introducing an additional loss function to the original KD loss operated on the feature extraction layer, as presented in Figure 1. In this section, we present the baseline model, the common KD method, and the proposed template-driven KD approach.

### 3.1. Baseline Models

We employed the widely used ResNet model [44] as a baseline model to evaluate our approach. The network architecture is based on an identity shortcut connection (residual connection) that skips one or more layers. The skip connection adds the input of a residual block to its output and passes it to the following residual block. ResNet won the ILSVRC 2015 challenge on the ImageNet database [45], with a top-5 error rate of 3.57%. The powerful representation ability of ResNet has motivated several computer vision tasks other than classification, such as object detection and face recognition [5]. In this work, we first evaluated the performance of three ResNet models—ResNet-34, ResNet-18, and ResNet-110 (without KD)—for periocular verification; all models are described in [44]. The first layer of ResNet-18 is a 7×7 convolutional layer followed by a 3×3 max-pooling layer. Then it stacks four convolutional groups. Each group consists of two residual blocks. Each block consists of two 3×3 convolutional layers with 64, 128, 256, and 512 filters for layers in groups 1, 2, 3, and 4, respectively. The last layer of the network is the average pooling layer (produced 512 features vector), followed by the classification layer. ResNet-34 has the same form as ResNet-18, with 3, 4, 6, and 3 residual blocks in groups 1, 2, 3, and 4, respectively. The number of trainable parameters in ResNet-34 and ResNet-18 is 21.3 million (m) and 11.8 m, respectively. Additionally, ResNet proposed the use of a small filter size of (32, 16, 8) with three groups of residual blocks to create a compact version of the ResNet model (ResNet-110) designed for the CIFAR-10 database with fewer trainable parameters. The used ResNet-110 in this work contains 1.8 m trainable parameters.

In this work, we deployed ResNet-34 and ResNet-18 as teacher networks and the compact ResNet-110 as a student, as well as a stand-alone baseline. In order to adopt these models for periocular verification, we modified the number of classes in the classification layer to match the number of identities in our training dataset. We applied transfer learning on the models ResNet-34 and ResNet-18 pretrained on the ImageNet dataset [45] by fine-tuning the last group of residual blocks on images from our training dataset with the softmax classifier. During the test phase, the softmax classifier was removed, and the features *f* were extracted from the last layer, with dimension 512×1×1.

The number features in the last layer of the ResNet-110 model is 64×1×1. In order to enable KD on the template level, we introduced an additional fully connected layer (before the classification layer) of size 512 to the ResNet-110 model to match the size of the embedding template of the teacher model. We trained this model from scratch on images from our training dataset. The model was trained first using the softmax classifier for baseline evaluation. During the test phase, the features *f* were extracted from the last fully connected layer. Using the exact training setups, we trained and evaluated the ResNet-110 model with the conventional (vanilla) KD approach. Finally, we trained and evaluated the ResNet-110 model with our template-driven KD.

### 3.2. Knowledge Distillation

KD is a technique to improve the performance and generalizability of smaller models by transferring the knowledge learned by a cumbersome model (teacher) to a single small model (student). The key idea is to guide the student model to learn the relationship between different classes discovered by the teacher model that contains more information beyond the ground-truth labels [40]. Suppose we have teacher model *T*, student model *S*, and training dataset X,Y∈D, where *X* is the training images and *Y* is their class labels. The output of the teacher model for any input $x_{i}\in X$ is a vector of class probabilities $P^{T}$ computed for each class using the softmax function by converting the logits, $z^{T}$, into probabilities that sum to one $P^{T}\left(x\right)=softmax\left(z^{T}\right)$. Specifically, the probability $p_{i}$ of class *i* is computed by comparing $z_{i}$ with other logits as given:

(1) $$p_{i}=\frac{exp(z_{i})}{\sum_{j=1}^{N}exp\left(z_{j}\right)}.$$

This probability distribution will have a high probability value of $p_{i}$ for the correct class $y_{i}\in Y$, with all other class probabilities close to zero. Thus, it does not provide more valuable information than ground-truth labels. Therefore, Hinton et al. [40] proposed to scale the logits using a temperature parameter t>1 before applying the softmax function. Thus, the teacher model can produce a softer distribution of the class probabilities, which provides more valuable information about classes similar to the predicted class. In this case, the output of the teacher model is $P_{s}^{T}\left(x\right)=softmax\left(z^{T}/t\right)$ and the probability $p_{i}$ of class *i* is given as:

(2) $$p_{i}=\frac{exp(z_{i}/t)}{\sum_{j=1}^{N}exp\left(z_{j}/t\right)}.$$

Similarly, the student model *S* can produce a soft class probability distribution using the temperature parameter *t*, $P_{s}^{S}\left(x\right)=softmax\left(z^{S}/t\right)$. The final loss for the student model is a weighted sum of two loss functions, cross-entropy loss $L_{ce}$ and Kullback–Leibler divergence (KLD) loss $L_{kld}$, as follows:

(3) $$L_{KD}=\alpha *L_{ce}\left(Y,P^{S}\left(x\right)\right)+\beta *t^{2}*L_{kld}\left(P_{s}^{S}\left(x\right),P_{s}^{T}\left(x\right)\right),$$

where *Y* is the ground-truth label, $P^{S}\left(x\right)$ is the standard softmax output produced by the student, $P_{s}^{S}\left(x\right)$ is the parameterized softmax output produced by the student, $P_{s}^{T}\left(x\right)$ is the parameterized softmax output produced by teacher, and α and β are the loss weight hyper-parameters. Since the gradients of the $L_{ce}$ loss are smaller than the gradients of the $L_{kld}$ where the logits used for $L_{kld}$ are divided by *t*, $L_{kld}$ is multiplied by $t^{2}$ as suggested by Hinton et al. [40].

### 3.3. Template-Driven Knowledge Distillation

Recently, a number of approaches have been proposed to improve the conventional vanilla KD method by either introducing an additional loss function to the KD loss [26] or by forcing the student to learn from multilayers instead of learning only from the logits of the last layer [46]. One of the drawbacks of softmax loss used in KD is that it does not explicitly optimize the embedded feature representation (template) needed for biometric verification. The template produced by the student model is less informative than the embedded template produced by the teacher model due to the shallow architecture of the student model. Therefore, in this work we propose to enhance the performance of the student model in the KD framework by driving it to learn the embedding template from the teacher model. In order to achieve this, we introduce an additional loss function to the original KD loss operated on the feature extraction layer. We evaluated the performance of the distilled model using two different loss functions: MSE and cosine embedding loss. Formally, the MSE is defined as follows:

(4) $$l_{mse}=1-\frac{1}{n}\Sigma_{i=1}^{n}{\left(\Phi_{t}^{S}{\left(x\right)}_{i}-\Phi_{t}^{T}{\left(x\right)}_{i}\right)}^{2},$$

where $\Phi_{t}^{S}$ and $\Phi_{t}^{T}$ are is the templates obtained from the last fully connected layer of student and teacher models, respectively, and *n* is the size of the template. The final template KD loss, in this case, for the student model can be defined as:

(5) $$\begin{array}{c} L_{KD}=\alpha *L_{ce}\left(Y,P^{S}\left(x\right)\right)+\beta *t^{2}*L_{kld}\left(P_{s}^{S}\left(x\right),P_{s}^{T}\left(x\right)\right)\\ +\beta *l_{mse}\left(\Phi_{t}^{S}\left(x\right),\Phi_{t}^{T}\left(x\right)\right), \end{array}$$

where $L_{ce}$ and $L_{kld}$ are the vanilla KD loss defined in Equation (3). In additional to MSE loss, we propose to evaluate the template distillation using cosine embedding loss defined as follows:

(6) $$loss_{cosine}=1-\frac{1}{n}\Sigma_{i=1}^{n}cos(\Phi_{t}^{S}{\left(x\right)}_{i},\Phi_{t}^{T}{\left(x\right)}_{i}).$$

The student loss using cosine distance for template KD can be defined as follows:

(7) $$\begin{array}{c} L_{KD}=\alpha *L_{ce}\left(Y,P^{S}\left(x\right)\right)+\beta *t^{2}*L_{kld}\left(P_{s}^{S}\left(x\right),P_{s}^{T}\left(x\right)\right)\\ +\beta *l_{cosine}\left(\Phi_{t}^{S}\left(x\right),\Phi_{t}^{T}\left(x\right)\right). \end{array}$$

### 3.4. Training Paradigm

The training paradigm of the proposed template-driven KD approach can be described in a step-wise algorithmic manner as follows: (1) Given a high-performing teacher model *T* and compact student model *S*, a batch of *n* training samples $\left(x_{i},y_{i}\right)$ is passed into the teacher and then the student model. $x_{i}\in X$ are periocular images of size 224×224 pixels and $y_{i}\in Y$ are their corresponding identity labels. (2) The outputs of the teacher model, that is, the predicted identity label $P_{s}^{T}\left(x\right)$ and the feature embedding $\Phi_{t}^{T}\left(x\right)$, are used to calculate the loss function defined in Equation (7) for the student model. (3) The gradient descent method, stochastic gradient descent (SGD), is utilized to calculate the derivatives of the loss function with respect to the student model weights. (4) These weights are updated with the backpropagation. (5) Steps 1 to 4 are repeated until the student model is converged. It should be noted that the backpropagation is used only to update the weight of the student model. The teacher model is pretrained, and all its weights are frozen during the KD training paradigm.

## 4. Experimental Setup

This section presents the implementation details and evaluation settings used in this work.

### 4.1. Periocular Database for Evaluation

We evaluated our proposed approach on a semi-public internal dataset of periocular images captured using two different smartphones—iPhone 5S and Nokia Lumia 1020. We chose this database as it provides a realistic cross-device evaluation protocol that reflects the real use of such technology where the sensor that captures the reference might be different from the one capturing the probe. The database contains 152 unique periocular identities captured captured under mixed illumination settings with external illumination from sunlight and illumination from artificial room light in day-light. The periocular images were captured using the rear camera of the smartphones in a semi-cooperative manner (assisted capture setting). For each identity, a number of multiple samples were captured, resulting in a total set of 6642 images (3341×2 smartphones).

**Figure 2:**
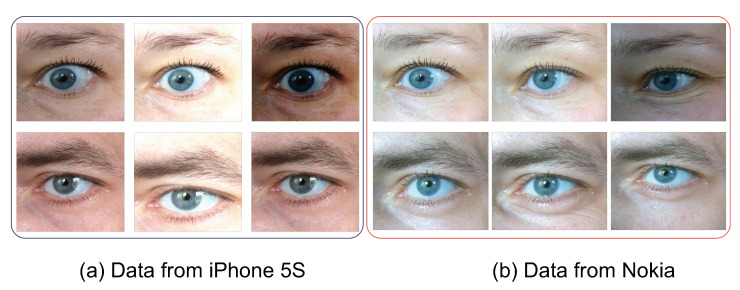
Sample images from the periocular database employed.

The periocular images in the database were captured to mimic everyday appearance variations that include make-up (e.g., the presence of mascara) and non-uniform illumination. The images also present a set of degradation due to motion blur and eye blinking, as encountered in everyday situations. Given that the images were captured in cross-illumination setting and with different cameras (iPhone 5S with 12 Mp and Nokia Lumia 1020 with 41 Mp), we first localized the periocular region using a Haar-cascade-based eye region detector. Further, the data were manually curated to eliminate any inconsistent segmentation in order to have only the periocular region. Given the nature of the images as illustrated in Figure 2, the challenging nature of periocular images could be deduced in cross-device verification settings. A careful observation Figure 2 reveals the variation in terms of appearance in different smartphones and the factors of degradation that were not constant across the phones/subjects.

For the sake of the experimental evaluation, we further divided the whole dataset of 152 unique periocular identities as a disjoint set of training and training with no identities in common. We chose the first 100 identities for the training dataset and the rest of 52 unique identities as a testing set to report our experimental results in the rest of this work.

### 4.2. Baseline and KD Training

The presented models in this work use ResNet [44] as baseline architecture. Our choice of ResNet as the backbone was based on the accuracy achieved by the recent biometric solutions utilizing ResNet as a feature extraction model [5,6,47]. ResNet-34 and ResNet-18 were trained with a batch size of 128, and the ResNet-110 model was trained with a batch size of 16. All models were trained using an SGD optimizer with Nesterov momentum 0.9. The initial learning rates were set to γ=0.001 and γ=0.1 for teacher and student models, respectively, and it was dropped by a factor of 0.1 after epoch 10 for the ResNet-110 model. We resized the training and testing images to 224×224 pixels to match the ResNet model input layer size. During the training, we augmented the training data by applying horizontal and vertical random shifting by up to 20% of the image width and/or height. ResNet-34 and ResNet-18 were fine-tuned for 5 epochs and ResNet-110 was trained for 25 epochs. ResNet-110 in conventional and template-driven KD approaches was trained using the exact ResNet-110 (without KD) training setup.

We followed the common choice for the knowledge distillation hyper-parameters [40,48,49] with temperature t>=4, α=0.9, and β=0.1.

### 4.3. Evaluation Protocols and Metrics

We evaluated the verification performance with cosine distance for comparison. The result is reported first for the models ResNet-34, ResNet-18, and ResNet-110, without applying KD. Additionally, we report the result of the conventional KD approach with KLD loss using the ResNet-110 model as the student with either ResNet-34 or ResNet-18 as the teacher, denoted as ResNet-110_KD34 and ResNet-110_KD18 respectively. Moreover, we demonstrate the performance of the proposed template-driven KD by reporting the results of using the ResNet-110 model as the student with either ResNet-34 or ResNet-18 as the teacher. These are denoted as ResNet-110_KD34MSE and ResNet-110_KD18MSE when the MSE loss is used, and as ResNet-110_KD34COS and ResNet-110_KD18COS when the cosine embedding loss is used.

For each of the settings, we investigated the verification performance under three different evaluation scenarios, defined as follows:

- iPhone verification scenario: The reference and the probe images were acquired using the camera of the iPhone smartphone.
- Nokia verification scenario: Similar to the previous scenario, the reference and the probe images were acquired using a Nokia smartphone.
- Cross-smartphone verification scenario: The reference and probe images were captured using two different smartphone cameras, where the reference images were captured using an iPhone camera and the probe images were captured using a Nokia camera.

The verification performance is reported using receiver operating characteristic (ROC) curves, area under the curve (AUC), FNMR at fixed FMR (FMR10: the lowest FNMR for FMR ≤ 10%, and FMR100: the lowest FNMR for FMR ≤ 1%), and equal error rate (EER).

### 4.4. Computational Efficiency

Table 1 presents the detailed analysis of the computational efficiency for each of the employed ResNet models. The computational efficiency of the deep learning approach depends on the number of trainable parameters and the inference latency. All evaluations were performed using PyTorch framework (Version 1.4) running on Linux OS with an Intel(R) Xeon(R) Gold 6130 CPU 2.10 GHz processor. ResNet-110 was the smallest model, with 1.8 m trainable parameters. The teacher models, ResBet-34 and ResNet-18, contained 21.3 m and 11.2 m trainable parameters. Regarding the inference time, ResNet-18 had the lowest latency of 0.007 ms. Note from Table 1 that the compact model (ResNet-110) had a higher latency than both ResNet-18 and ResNet-34. This is mainly because of the large number of convolutional layers in ResNet-110 (110 convolutional layers), as we discussed in Section 3, in comparison to 18 layers in ResNet-18 and 34 layers in ResNet-34. As the size of the model makes up the largest deployment challenge, the focus of this work was to enhance the accuracy of the small ResNet-110 model using the proposed template-driven KD.

## 5. Results

**Figure 3:**
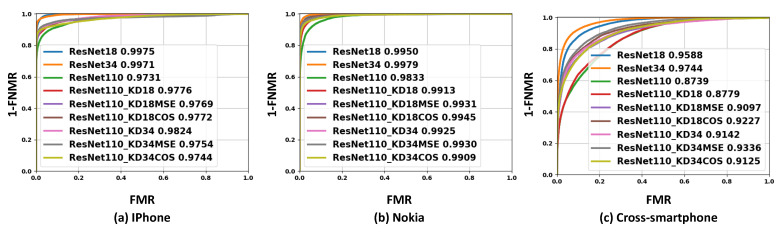
The achieved ROC curves for different experimental settings. For each experimental setting, the number next to the model label is the achieved AUC. Note the improvement in the ResNet-110 verification performance using our proposed template-driven KD approach.

The verification performances of the different experimental settings are presented in Figure 3 along with the EER, FMR10, and FMR100 values in Table 2. Figure 3a–c shows the achieved ROC curves of the iPhone verification scenario (a), the Nokia verification scenario (b), and the cross-smartphone verification scenario (c).

### 5.1. Baseline

The first three rows of Table 2 present the achieved verification performance by ResNet-34, ResNet-18 and ResNet-110 (without KD). As expected, ResNet-34 and ResNet-18 achieved higher verification performances than the compact model ResNet-110 for different verification scenarios. In the cross-smartphone verification scenario, the best achieved EER was 8.17% by the ResNet-34 model, which indicates the high generalizability of this model. In this case, ResNet-18 achieved a competitive verification performance to ResNet-34 with 11.04% EER. ResNet-110 achieved the lowest verification performance, with 22.16% EER. For the iPhone verification scenario, the best achieved EER was 2.51% by ResNet-18 followed very closely by ResNet-34 with 2.73% EER, then ResNet-110 with 8.83% EER. The best-achieved EER for the Nokia verification scenario was 2.18% by ResNet-34, followed by ResNet-18 with 3.04% EER and ResNet-110 with 6.94% EER. Further, it can be noticed that the verification performances dropped for all evaluated models when the reference and probe images were captured from different smartphones in comparison to the case where the probe and the reference images were captured from the same smartphone, as shown in Table 2. However, this degradation in the performance is a common issue for cross-smartphone verification scenarios, as reported in previous works [50].

### 5.2. Knowledge Distillation

The achieved results by the conventional KD approach are presented in Table 2 and Figure 1. It can be observed from the Table 2 that introducing conventional KD to the ResNet-110 training process (ResNet-110_KD18 and ResNet-110_KD34) highly improved the verification performances for different verification scenarios. For the cross-smartphone verification scenario, the EER was reduced from 22.16% to 21.87% and 16.61% when the knowledge was distilled from teacher ResNet-18 and ResNet-34, respectively. Similar improvements in verification performance can also be seen for Nokia and iPhone verification scenarios.

### 5.3. Template-Driven Knowledge Distillation

The results presented in Table 2 and Figure 3 illustrate that the verification performances of the ResNet-110 model were significantly improved by applying our proposed template-driven KD approach on the ResNet-110 training process in comparison to the case when ResNet-110 was trained with the conventional KD approach. For all verification scenarios, it can be observed from Table 2 that ResNet-110 trained with either cosine (ResNet-110_KD18COS and ResNet-110_KD34COS) or MSE embedding loss (ResNet-110_KD18MSE and ResNet-110_KD34MSE) outperformed the same model trained without KD and with the conventional KD approach. For the cross-smartphone verification scenario, the EER was improved from 21.87% (ResNet-110_KD18) to 17.53% (ResNet-110_KD18MSE) using MSE embedding loss and to 15.87% (ResNet-110_KD18COS) using cosine embedding loss. A similar improvement in the verification performance was achieved using ResNet-34 as teacher with template-driven KD, where the best achieved EER, in this case, for the cross-smartphone scenario, was 14.69% (ResNet-110_KD34MSE). For both Nokia and iPhone verification scenarios, the achieved verification performance by ResNet-110 using our proposed template-driven KD was significantly improved and even very close to the deeper models ResNet-34 and ResNet-18, as shown in Table 2 and Figure 3.

In comparison to the recent works that focused on providing compact periocular recognition models, our proposed model (1.8 m parameters) outperformed the DenseNet-20 (1.1 m parameters) proposed by [43] on the most challenging settings, cross-smartphone. The proposed DenseNet-20 by [43] was trained with conventional KD, where the compact model was trained to learn a similar classification decision to the teacher model. Different from conventional KD approaches [43], our proposed template-driven KD optimizes the student model so that it learns to output similar embeddings (template) to the teacher model, which is the requirement for a high-performing biometric template. The conventional classification KD in [43] is conceptually similar to our baseline ResNet-100_KD18 and ResNet-110_KD34. In this case, the best reported EER by [43] in cross-device verification was 15.54%, and the best reported one by our approach was 14.69%. Towards the goal of efficient periocular recognition, Almadan et al. [51] utilized conventional KD to train MobileNet-V2 [52] (3.5 m parameters), MobileNet-V3 [53] (2.5 m parameters), ResNet-20 [44] (1.3 m parameters), and ShuffleNetV2-50 [54] (1.4 m parameters) with ResNet50 [44] as a teacher model. These models were trained and evaluated on VISOB [55] and UFPR datasets [56]. Among the evaluated models, MobileNet-V2 (3.5 m parameters) achieved the lowest EER: 5.21% on VISOB and 5.38% on the UFPR dataset. Meanwhile, the ShuffleNetV2-50 model in [51], with a comparable size (1.4 m parameters) to our light model, scored an EER of 23.41% on VISOB and 22.52% on the UFPR datasest, although the results in [51] cannot be compared directly to the results in this work as they were evaluated on a different benchmark.

## 6. Conclusions

This work was motivated by the need for compact biometric deep learning models for deployment on resource-critical devices. Such models aim to have the capability to extract highly distinctive templates, as the larger models (more learned parameters) do. KD has been used to map such knowledge by teaching a smaller student model to produce a similar output as a larger teacher model. However, given that KD is commonly optimized on the classification output of such models, the knowledge of extracting biometric templates from pre-classification layers might not be optimally transferred to the student model. Therefore, we proposed a novel template-driven KD approach that aims at teaching the template extraction knowledge to the student model. The proposed approach was evaluated on smartphone periocular verification in intra- and cross-device settings. The achieved results showed that when the targeted small model was trained with our template-driven KD approach, it consistently outperformed similar models trained without KD or with the conventional KD approach.

## Figures and Tables

**Table 1:** The inference time (in milliseconds) and the number of trainable parameters (in millions (m)) for each of the evaluated models.

Model	Inference Time	No. Trainable Parameters
ReseNet-110	0.015 ms	1.8 m
ResNet-18	0.007 ms	11.2 m
ResNet-34	0.008 ms	21.3 m

**Table 2:** Performance obtained for different experimental settings. The first three rows of the table present the achieved result for the three evaluated models (without using KD), where the smallest model (ResNet-100) performed the worst. The next three rows of the table present the achieved verification performance by including KD in the training process using ResNet-18 as a teacher with conventional KD loss, and both the template-driven KD with MSE and cosine (COS) embedding loss. The last three rows present the achieved KD verification performance using ResNet-34 as a teacher with conventional KD, and both the template-driven KD with MSE and cosine embedding loss. The enhanced performance by the proposed method in comparison to the conventional KD is illustrated over all experimental setups. The lowest EER, FMR10 and FMR100 for each of the verification scenarios (iPhone, Nokia and Cross-Smartphone) achieved by ResNet-100 are in bold.

Model	Teacher	iPhone			Nokia			Cross-Smartphone		
		EER	FMR10	FMR100	EER	FMR10	FMR100	EER	FMR10	FMR100
ResNet-18	-	0.0251	0.0032	0.0557	0.0304	0.0108	0.0776	0.1104	0.1216	0.5171
ResNet-34	-	0.0273	0.0071	0.0431	0.0218	0.0033	0.0434	0.0871	0.0767	0.3267
ResNet-110	-	0.0883	0.0839	0.1984	0.0694	0.0473	0.2298	0.2216	0.3945	0.6932
ResNet-110_KD18	ResNet-18	0.0713	0.0547	0.1401	0.0471	0.0198	0.1284	0.2187	0.3619	0.6872
ResNet-110_KD18MSE	ResNet-18	0.0632	0.0500	0.1401	0.0353	0.0114	0.0763	0.1753	0.2539	0.5752
ResNet-110_KD18COS	ResNet-18	0.0702	0.0571	0.1171	**0.0330**	**0.0107**	0.0724	0.1587	0.2215	0.5051
ResNet-110_KD34	ResNet-34	0.0651	0.0530	0.1153	0.0413	0.0227	0.0844	0.1661	0.2321	0.5494
ResNet-110_KD34MSE	ResNet-34	**0.0614**	**0.0470**	**0.1136**	0.0375	0.0193	**0.0692**	**0.1469**	**0.2010**	**0.4984**
ResNet-110_KD34COS	ResNet-34	0.0775	0.0711	0.1161	0.0457	0.0265	0.0962	0.1725	0.2580	0.5368

## Data Availability

Not applicable.

## Abbreviations

The following abbreviations are used in this manuscript:

KD	knowledge distillation
MSE	mean squared error
FNMR	false non-match rate
FMR	false match rate
EER	equal error rate
